# Estradiol Attenuates the Severity of Primary *Toxoplasma gondii* Infection-Induced Adverse Pregnancy Outcomes Through the Regulation of Tregs in a Dose-Dependent Manner

**DOI:** 10.3389/fimmu.2018.01102

**Published:** 2018-05-18

**Authors:** Jingfan Qiu, Rong Zhang, Yanci Xie, Lijuan Wang, Ke Ge, Hao Chen, Xinjian Liu, Jiangping Wu, Yong Wang

**Affiliations:** ^1^Key Laboratory of Pathogen Biology of Jiangsu Province, Department of Pathogen Biology, Nanjing Medical University, Nanjing, China; ^2^Xuanwumen Community Health Service Center of Xuanwu District, Nanjing, China; ^3^The First Clinical Medical College, Nanjing Medical University, Nanjing, China; ^4^Nanjing Maternity and Child Health Care Hospital Affiliated to Nanjing Medical University, Nanjing, China; ^5^Key Laboratory of Infectious Diseases, School of Public Health, Nanjing Medical University, Nanjing, China; ^6^State Key Laboratory of Reproductive Medicine, Nanjing Medical University, Nanjing, China

**Keywords:** estradiol, *Toxoplasma gondii*, regulatory T cells, programmed death-1, abortion, apoptosis

## Abstract

Estradiol (E2) plays a crucial and intricate role during pregnancy to mediate several aspects of the pregnancy process. A perplexing phenomenon in congenital toxoplasmosis is that the severity of *Toxoplasma gondii* (*T. gondii*)-mediated adverse pregnancy outcome is closely related with time of primary maternal infection during pregnancy. In this study, the results showed that *T. gondii* infection in early pregnancy was more likely to induce miscarriage in mice than in late pregnancy, which may be related to inflammation of the maternal–fetal interface. Meanwhile, the *T. gondii* infection-induced-apoptotic rate of Tregs was higher and the expression of programmed death-1 (PD-1) on Tregs was lower in early pregnancy than in late pregnancy. As the level of E2 in mouse serum gradually increased with the development of pregnancy, we proposed that E2 may contribute to the discrepancy of Tregs at different stages of pregnancy. Thus, we investigated *in vitro* and *in vivo* effects of E2 in regulating Tregs. We found that E2 *in vitro* could protect Tregs against apoptosis and upregulate the expression of PD-1 on Tregs in a dose-dependent manner through ERα. Likewise, the simulated mid-pregnancy level of E2 in nonpregnant mice also alleviated the *T. gondii* infection-induced apoptosis of Tregs and potentiated the PD-1 expression on Tregs. Therefore, in the pathogenesis of *T. gondii*-induced abnormal pregnancy, E2 helped maintain the immune balance and improve the pregnancy outcome through regulating Tregs. This finding illustrates the intricate working of hormone–immune system interaction in infection-induced abnormal pregnancy.

## Introduction

*Toxoplasma gondii* (*T. gondii*), an opportunistic intracellular parasitic protozoon, can cause disease in the developing fetus (1, 2). It is the causative agent of congenital toxoplasmosis. Maternal infection of *T. gondii* during the first trimester of human pregnancy could result in severe congenital toxoplasmosis, while the infection that occurs at the late pregnancy usually results in low rate of ill newborns (3, 4). Interestingly, the parasites were seldom found in the maternal uterus or placenta with *T. gondii* infection at the early stage of pregnancy (2). Such *T. gondii*-induced-impaired pregnancy outcome might be associated with a high inflammatory response leading to cell apoptosis and necrosis of the implantation sites (5). The *T. gondii*-induced adverse pregnancy outcomes depend on the timing of primary infection during pregnancy, yet the cellular and molecular mechanisms involved are not fully understood.

Pregnancy is a state of immunological tolerance. Several specialized mechanisms were proposed as having contributed to such a tolerance. Among them, CD4^+^CD25^+^ regulatory T cells (Tregs) were regarded as important players in maintaining normal pregnancy (6). Reduced numbers of Tregs correlated with the immunological rejection of the fetus, which could be prevented by adoptively transferring Tregs from normal pregnant animals (7, 8). Infection of mice with *T. gondii* triggers a powerful Th1 immune response and a reduction in Tregs (9, 10). Studies have shown that Tregs exert their immune-protective function during pregnancy through a negative costimulatory molecule programmed death-1 (PD-1) (11). Liu et al. observed a decrease in the absolute numbers of PD-1^+^ Tregs in *T. gondii*-infected mice. Adoptive transfer of PD-1^+^-abundant Tregs from fetal–maternal interface of normal pregnant mice improved pregnancy outcome of *T. gondii*-infected mice (12). Our previous study demonstrated that mice treated with *T. gondii* excreted–secreted antigens (ESA) at the early stage of pregnancy induced the diminished quantity and impaired the suppressive capacity of Tregs, subsequently resulting in the abortion, while the treatment that occurs at the late pregnancy fails to cause it, due to the unimpaired capacity of Tregs (13). It is obvious that different pregnancy outcomes are associated with the number and function of Tregs after injection of *T. gondii* antigens in different periods of gestation. However, it remains unclear why a same injection of *T. gondii* ESA has different effects on Tregs at different stages of pregnancy.

During pregnancy, there are high levels of female sex hormones in serum. Among those hormones, the effects of estradiol (E2) (17-β-E2) on immune function have been generally reported (14). The ability of E2 to protect against autoimmune disease correlated with its ability to increase CD25^+^ cell number and upregulate Foxp3 expression *in vitro* and *in vivo* (15, 16). Tai P also found that E2, at physiological doses, stimulated the conversion of CD4^+^CD25^−^ T cells into CD4^+^CD25^+^ T cells which exhibited an enhanced Foxp3 expression *in vitro* (17). In addition, enhanced suppression was detected when Tregs were pretreated with E2 (18, 19). Together, these data suggest that E2 modulates Tregs by regulating their number and potentiating their function.

The mechanisms by which E2 modulates the number and function of Tregs have been reported in a few disease models. Although it is not demonstrated that E2 can reduce the apoptosis of Tregs (20), many studies have suggested that E2 can downregulate the apoptosis of certain cells, such as cardiomyocyte (21), endothelial cells (22), and skeletal myoblasts (23). Moreover, E2 is a potent driver of Tregs. It can promote the expression of PD-1 on Tregs. Polanczyk MJ demonstrated that E2 treatment increased PD-1 expression on Tregs. Both the expression of PD-1 and the suppression function of Tregs were reduced in ER KO mice (24). In experimental autoimmune encephalomyelitis (EAE) model, E2 induced protection against EAE by upregulating PD-1 expression on Tregs (25). Therefore, we speculate that the level of maternal E2 secretion at different stages of pregnancy might govern the number and function of Tregs *via* regulating the apoptosis and PD-1 expression of Tregs, thus contributing to *T. gondii*-induced different reproductive outcomes.

In this study, we hope to reveal the mechanism that the severity of *T. gondii*-induced adverse pregnancy outcome is influenced by the timing of primary infection during pregnancy through the aspects of Tregs. Given the effects of E2 on Tregs, we studied the specific mechanism by which E2 exerts immune-modulatory effects on Tregs. We found that a high level of E2 in late pregnancy alleviated the *T. gondii* infection-induced apoptosis of Tregs and potentiated the PD-1 expression on Tregs, thus helping maintain the immune balance and improve the pregnancy outcome. These results further shed light on the mechanisms by which the tolerance of pregnancy breaks down and abortion develops. Moreover, this finding provides new thoughts for the development of hormone-based immune disorder treatment (26).

## Materials and Methods

### Ethics Statement

This study received approval from the Animal Ethics Committee of Nanjing Medical University, and all animal experiments were conducted in accordance with the approved guidelines for the care and use of animals (Permit Number: NJMU/IACUC 1403005).

### Mice and Mating

Six-week-old C57BL/6 female and eight-week-old C57BL/6 male mice were purchased from the Model Animal Research Center of Nanjing University (Nanjing, China). The mice were housed in stainless steel cages and bred with free access to water and food under conditions of controlled temperature (22 ± 2°C) and humidity (50 ± 5%) with a 12/12-h light/dark cycle. The mice were acclimated for 1 week. Then, female and male mice were paired in the evening. A vaginal plug confirmed in the next morning was defined as day 0 of pregnancy (G0). Tachyzoites (5 × 10^4^) of *T. gondii* were intraperitoneally inoculated into pregnant mice at gestational days 5 (G5) and 15 (G15), respectively. The injection of the same volume of PBS was conducted as control. All the animals were sacrificed at 3 days post injection, and the pregnancy outcomes were observed. The necrosis and hemorrhage of fetuses and placentas were identified by visual observation. The abortion rate was calculated as the ratio of abortion sites to total implantation sites as described previously (13, 27). The abortion sites were identified by their shrunk size, necrotic, and hemorrhagic appearance compared with normal embryos and placenta. For the detection of E2 in the serum, the serum from pregnant mice during the entire course of pregnancy at gestation days 0, 5, 6, 7, 8, 9, 14, 15, 16, 17, and 18 (G0, G5, G6, G7, G8, G9, G14, G15, G16, G17, and G18) was collected and measured by ELISA using Mouse Estradiol ELISA Kit (Cusabio, Wuhan, China).

### *T. gondii* and ESA Preparation

*Toxoplasma gondii* tachyzoites of RH strain were preserved in liquid nitrogen and resuscitated in C57BL/6 mice by intraperitoneal inoculation. ESA were prepared as described previously (28). The ESA were treated with AffinityPak Detoxi-Gel Endotoxin Removing Gel (Thermo, Fairlawn, OH, USA) to remove endotoxin. Endotoxin levels in RH ESA were <0.1 EU/mg protein, as determined by a Limulus assay (Xiamen Limulus Reagent Co., Ltd., Xiamen, China). The ESA-containing fluid was then filtered through a 0.22-µm filter (Merck Millipore, Billerica, MA, USA), and protein concentrations of the filtrate were assayed by using the Bradford method.

### Histochemistry Analysis and Immunofluorescence (IF) Assays

Tissue sections of uteri from nonpregnant and pregnant mice were fixed in 4% paraformaldehyde–PBS, dehydrated in a graded series of alcohol solutions, embedded in paraffin, and then sectioned (4 µm). Then, the slides were stained with hematoxylin & eosin (H&E) and observed under a light microscope (Zeiss, Oberkochen, Germany). The images were taken using AxioVision Rel. 4.8 software (Zeiss).

For IF analyses, paraffin sections were deparaffinized and blocked with 5% bovine serum albumin (Sigma-Aldrich, St. Louis, MO, USA). In the case of single staining of Foxp3 and PD-1, the slides were incubated with anti-Foxp3 antibody (1:100, Servicebio, Wuhan, China) and anti-PD-1 antibody (1:100, proteintech, Wuhan, China), respectively, overnight at 4°C. The sections were then washed and incubated for 50 min at room temperature with corresponding secondary antibody (Cy3-conjugated anti-rabbit IgG, Alexa Fluor^®^ 488-conjugated anti-rabbit IgG; Servicebio). In the case of double staining of Foxp3 and PD-1, the slides were incubated with anti-Foxp3 antibody (1:200, Servicebio) and anti-PD-1 antibody (1:100, R&D Systems, Minneapolis, MN, USA) overnight at 4°C. The sections were then washed and incubated for 50 min at room temperature with secondary antibody (Cy3-conjugated anti-rabbit IgG, FITC-conjugated anti-goat IgG; Servicebio). DAPI was used to mark the nuclei. The slides were examined under a fluorescent microscopy (Nikon Eclipse C1, Nikon, Japan) and the images were taken by NIKON DS-U3 software (Nikon).

### Splenocyte Isolation and E2 Treatment

Splenocytes were harvested from 6-week-old C57BL/6 mice and cultured in a round-bottom 24 well plate (Corning, Costar) using complete RPMI 1640 medium at 37°C in 5% CO_2_ *in vitro*. Splenocytes (1 × 10^6^/ml) were treated with E2 (0, 10^−10^, 10^−8^, and 10^−6^ M, Sigma-Aldrich) for 24, 48, 72, and 96 h, respectively, and then harvested for flow cytometric analysis. The culture supernatants were collected after 96 h and frozen at −80°C for cytokine detection. The levels of IFN-γ, IL-10, and TGF-β1 were measured by ELISA according to the instructions of the manufacturer (Dakewe, Shenzhen, China).

### Estrogen Receptor (ER) Agonist and Antagonist Treatment

Estrogen receptor-α agonist 1,3,5-Tris(4-hydroxyphenyl)-4-propyl-1H-pyrazole (PPT) and ER antagonist, fulvestrant (ICI 182,780), were, respectively, used to assess the role of ERα in inhibiting the apoptosis of Tregs. In ERα agonist assay, splenocytes (1 × 10^6^/ml) were pre-incubated with E2 (10^−8^ M) and different concentrations of PPT (10^−10^, 10^−8^, and 10^−6^ M), respectively, for 24 h. Then, ESA (10 µg/ml) were added to the splenocyte cultures for 48 h and harvested for flow cytometric analysis. In ER antagonist assay, splenocytes (1 × 10^6^/ml) were pre-incubated with ICI 182,780 (10^−5^ M) for 1 h. Then, E2 (10^−8^ M) was added into the culture. After 24 h, ESA (10 µg/ml) were added to the splenocyte culture for 48 h and then harvested for flow cytometric analysis.

To evaluate the role of ERα in enhancing the expression of PD-1 on Tregs, the expression of PD-1 on Tregs was analyzed with the treatment of PPT and fulvestrant (ICI 182,780). As for ICI 182,780 and E2-treated group, splenocytes (1 × 10^6^/ml) were pre-incubated with ICI 182,780 (10^−5^ M) for 1 h and then incubated with E2 (10^−8^ M) for 48 h. In other groups, splenocytes (1 × 10^6^/ml) were treated separately with E2 (10^−8^ M), different concentrations of PPT (10^−10^, 10^−8^, 10^−6^ M), ICI 182,780 (10^−5^ M), and ESA (10 µg/ml) for 48 h. After 48-h treatment, the splenocytes were harvested for flow cytometric analysis.

### E2 *In Vivo* Administration in Nonpregnant Mice

Estradiol (800 ng/day, Sigma-Aldrich) was subcutaneously injected into the female 6-week-old nonpregnant C57BL/6 mice daily for 2 weeks. After a 2-week injection of E2, the level of E2 in mouse serum was detected using Mouse Estradiol ELISA Kit (Cusabio, Wuhan, China). Mice were euthanized after a 2-week treatment and splenocytes were collected. The percentage of apoptotic Tregs and the expression of PD-1 on Tregs in spleens and inguinal lymph nodes were detected by flow cytometry.

### Flow Cytometry

For the analysis of the apoptosis of CD4^+^CD25^+^ T cells, splenocytes were stained with PE-conjugated antibody against mouse CD4 and APC-conjugated antibody against mouse CD25 (eBioscience, San Diego, CA, USA), washed, and then stained with FITC-labeled Annexin V and 7AAD (BD Biosciences, San Jose, CA, USA). CD4^+^CD25^+^ T cells were gated for Annexin V/7AAD analysis. CD4^+^CD25^+^ T cells in early apoptosis (Annexin^+^7AAD^−^) were in the lower right quadrant. Live cells (Annexin^−^7AAD^−^) were in the lower left quadrant. Dead cells (Annexin^+^7AAD^+^) were in the upper right quadrant.

For the analysis of the PD-1 expression on CD4^+^CD25^+^Foxp3^+^ T cells, splenocytes were incubated with FITC-conjugated antibody against mouse CD4 (BD Biosciences), APC-conjugated antibody against mouse CD25 (eBioscience), and PE/Cy7-conjugated antibody against mouse PD-1 (BioLegend, San Diego, CA, USA) at 4°C in the dark for 30 min and then were washed. For intracellular staining of Foxp3, the cells were fixed and permeabilized in 1 × Fix/Perm buffer (eBioscience) for 1 h and then were washed twice according to the manufacturer’s instructions. The cells were collected and incubated with Mouse FcR Block antibody (BD Biosciences) at 4°C for 15 min and then stained with PE-conjugated antibody against mouse Foxp3 (eBioscience). After washing twice with PBS, the cells were resuspended for analysis by flow cytometry.

For the analysis of the caspase-3 expression on CD4^+^CD25^+^ T cells, splenocytes were stained with FITC-conjugated antibody against mouse CD4 (BD Biosciences) and APC-conjugated antibody against mouse CD25 (eBioscience), washed, and incubated in Cytofix/Cytoperm solution (BD Biosciences) for 20 min on ice. Then, washed twice with Perm/Wash buffer (BD Biosciences) at room temperature, stained with PE-labeled antibody against mouse caspase-3 (BD Biosciences) for 30 min at room temperature, and then analyzed by flow cytometry.

For the analysis of the Bcl-2 expression on CD4^+^CD25^+^Foxp3^+^ T cells, splenocytes were incubated with FITC-conjugated antibody against mouse CD4 (BD Biosciences), APC-conjugated antibody against mouse CD25 (eBioscience) at 4°C in the dark for 30 min, and then washed. For intracellular staining of Foxp3 and Bcl-2, the cells were fixed and permeabilized in 1 × Fix/Perm buffer (eBioscience) for 1 h and then washed twice according to the manufacturer’s instructions. The cells were collected and incubated with Mouse FcR Block antibody (BD Biosciences) at 4°C for 15 min and then stained with PE-conjugated antibody against mouse Foxp3 (eBioscience) and PE-Cy7-conjugated antibody against mouse Bcl-2 (Biolegend). After washing twice with PBS, the cells were resuspended for analysis by flow cytometry.

### Statistical Analyses

SPSS software was used to determine the statistical significance of differences in the means of experimental groups. Data of two groups were analyzed for statistical significance with Student’s *t*-test. Multiple comparisons were made by using one-way ANOVA.

## Results

### *T*. *gondii* Infection in Early Pregnancy Induced Inflammation in the Maternal–Fetal Interface of Mice

**Figure 1:**
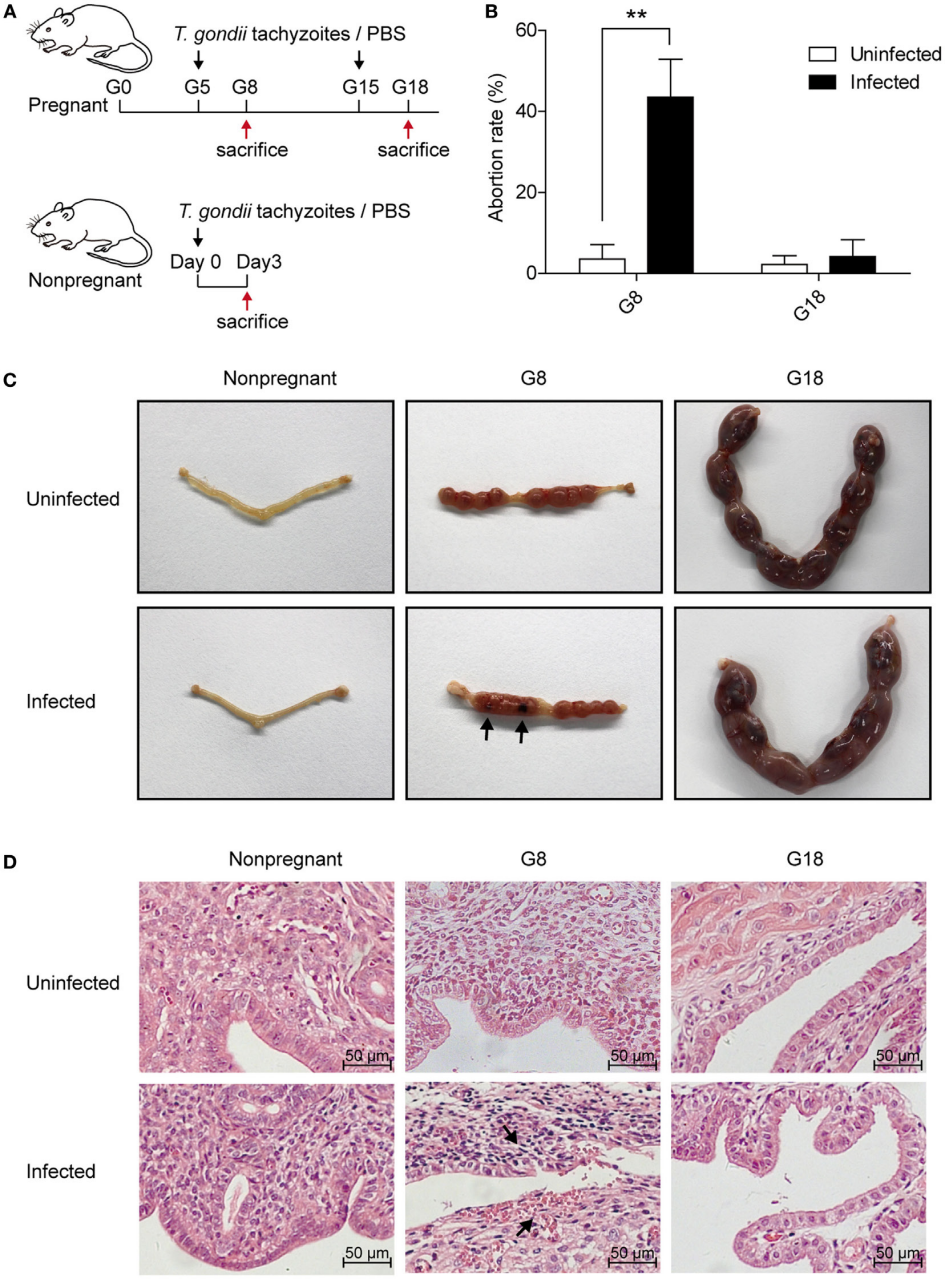
The discrepancy of pregnancy outcomes in mice infected with *Toxoplasma gondii* tachyzoites at different stages of pregnancy. **(A)** Time schedule for *T. gondii* infection. Nonpregnant and pregnant mice at gestational days 5 (G5) and 15 (G15) were injected with *T. gondii* tachyzoites or PBS, respectively. All the animals were sacrificed at 3 days post infection (nonpregnant, G8 and G18). **(B)** The abortion rates of pregnant mice at gestational days 8 (G8) and 18 (G18) with *T. gondii* tachyzoite or PBS injection. The abortion rate was calculated as the ratio of abortion sites to the total number of implantation sites. Data are expressed as the means ± SD of five mice for each group from one experiment representative of three independent experiments. **P < 0.01. **(C)** Representative pictures of uteri from nonpregnant and pregnant mice at G8 and G18 with *T. gondii* tachyzoite or PBS injection. Fetuses and placentas of early pregnant mice (G8) suffered necrosis and hemorrhage after *T. gondii* infection (black arrow), while late pregnant mice (G18) did not exhibit any adverse pregnancy outcomes after infection. **(D)** Representative histopathological sections with H&E staining of the maternal–fetal interface from mice as mentioned above. The maternal–fetal interface of early pregnant mice (G8) displayed obvious hemorrhage and inflammation with *T. gondii* infection at G5 (black arrow), while pregnant mice (G18) with infection at G15 did not exhibit any adverse pregnancy outcomes. Scale bars, 50 µm.

To investigate the discrepancy of pregnancy outcomes with *T. gondii* infection at different stages of gestation, we first, respectively, injected nonpregnant, early (G5) and late (G15) pregnant mice with the same number of tachyzoites or PBS intraperitoneally. Then, the mice were sacrificed after 3 days post infection (Figure 1A). The abortion rates of pregnant mice with *T. gondii* infection at early pregnancy (G5) were significantly higher than those of uninfected mice (*P* < 0.01) (Figure 1B). Fetuses and placentas of early pregnant mice suffered necrosis and hemorrhage after infection, with the abortion rate up to 43.5% (Figure 1C). However, there was hardly any visible fetal abnormality in pregnant mice with *T. gondii* infection at late pregnancy (G15). Uteri sections from nonpregnant and pregnant mice (G8, G18) with *T. gondii* infection were then stained with H&E (Figure 1D). Maternal–fetal interface of infected mice from early pregnant group behaved obviously hemorrhagic and inflammatory, but not from late pregnant group. This result confirmed that the difference in pregnancy outcome was closely related to the timing of primary *T. gondii* infection. *T. gondii* infection in early pregnancy was more likely to induce miscarriage in mice than in late pregnancy, which may be related to the inflammation of the maternal–fetal interface.

### *T*. *gondii* Infection in Early Pregnancy Induced Higher Levels of Apoptotic Rate and Lower Levels of PD-1 Expression on Tregs Than That in Late Pregnancy

**Figure 2:**
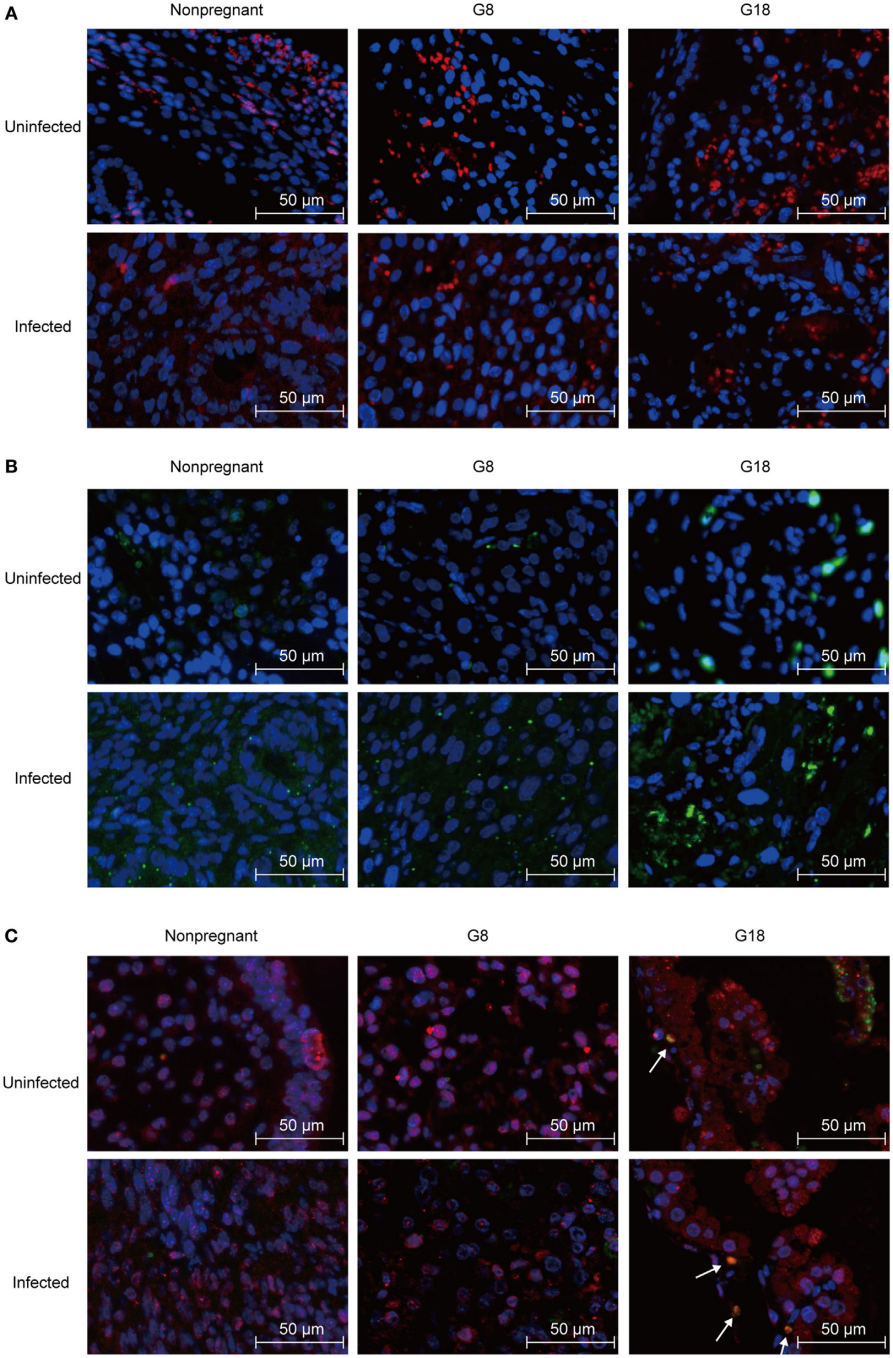
Expression of Foxp3 and programmed death-1 (PD-1) at the maternal–fetal interface of mice assessed by immunofluorescence staining. Nonpregnant and pregnant mice at G5 and G15 were injected with *Toxoplasma gondii* tachyzoites or PBS, respectively. All the animals were sacrificed at 3 days post infection (nonpregnant, G8 and G18). **(A)** Single staining of Foxp3^+^ cells. Blue, DAP1; red, Foxp3. Scale bars, 50 µm. **(B)** Single staining of PD-1^+^ cells. Blue, DAP1; green, PD-1. Scale bars, 50 µm. **(C)** Double staining of PD-1 and Foxp3. Blue, DAP1; green, PD-1; red, Foxp3. Scale bars, 50 µm. White arrows indicate PD-1^+^Foxp3^+^ cells.

It is generally believed that a diminished number of Tregs were associated with inflammation of maternal–fetal interface, and PD-1 could mediate the protective function of Tregs (8, 29). Thus, we analyzed Foxp3 and PD-1 expression in the maternal–fetal interface of *T. gondii*-infected mice. The same number of tachyzoites was, respectively, injected into the early (G5) and late (G15) pregnant mice and nonpregnant mice intraperitoneally. Then, the mice were sacrificed after 3 days post infection. Uteri sections from nonpregnant and pregnant mice (G8, G18) were stained with anti-Foxp3 and anti-PD-1 IF antibodies, respectively. The expression of Foxp3 in the maternal–fetal interface of mice in early pregnancy was lower than that in late pregnancy and higher than the expression of Foxp3 in endometria from nonpregnant mice (Figure 2A). This phenomenon appeared not only in *T. gondii*-infected group but also in uninfected group, yet the expression of Foxp3 in infected mice was lower than that in uninfected mice. Contrarily, PD-1 expression in maternal–fetal interface increased after infection with *T. gondii* tachyzoites (Figure 2B). In addition, the expression of PD-1 in the maternal–fetal interface of mice in early pregnancy was lower than that in late pregnancy. Double staining of PD-1 and Foxp3 showed that PD-1^+^Foxp3^+^ T cells increased in the maternal–fetal interface of late pregnant mice in uninfected and infected groups (Figure 2C). Moreover, some PD-1^+^Foxp3^−^ cells were also observed in these groups. These results suggested that the maternal–fetal interface of mice with *T*. *gondii* infection in late pregnancy displayed a higher level of Foxp3 and PD-1 expression than that in early pregnancy.

**Figure 3:**
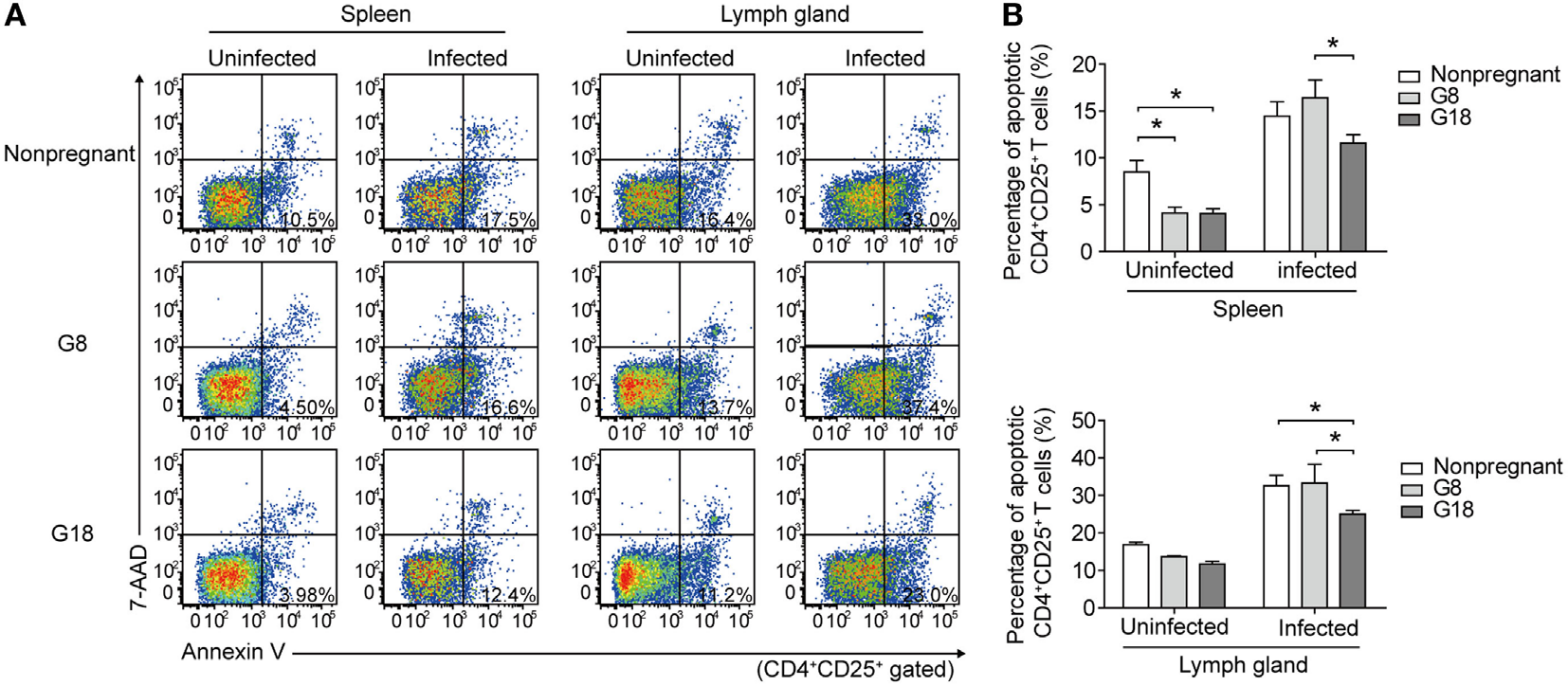
*Toxoplasma gondii* infection in early pregnancy induced higher levels of apoptotic rate of Tregs than that in late pregnancy. **(A)** The apoptotic rate of CD4^+^CD25^+^ T cells in spleens and inguinal lymph glands of the nonpregnant and pregnant mice (G8, G18) was evaluated by flow cytometry. The gating strategy for the identification of the mouse CD4^+^CD25^+^ T cell population is shown in Figure S1A in Supplementary Material. CD4^+^CD25^+^ T cells in early apoptosis (Annexin^+^7AAD^−^) were in the lower right quadrant. **(B)** The apoptotic rate of CD4^+^CD25^+^ T cells in spleens and inguinal lymph glands of the nonpregnant and pregnant mice (G8, G18) with *T. gondii* tachyzoite or PBS injection, respectively. Data are expressed as the means ± SD of five mice for each group from one experiment representative of two independent experiments. Significance was determined by one-way ANOVA. *P < 0.05.

**Figure 4:**
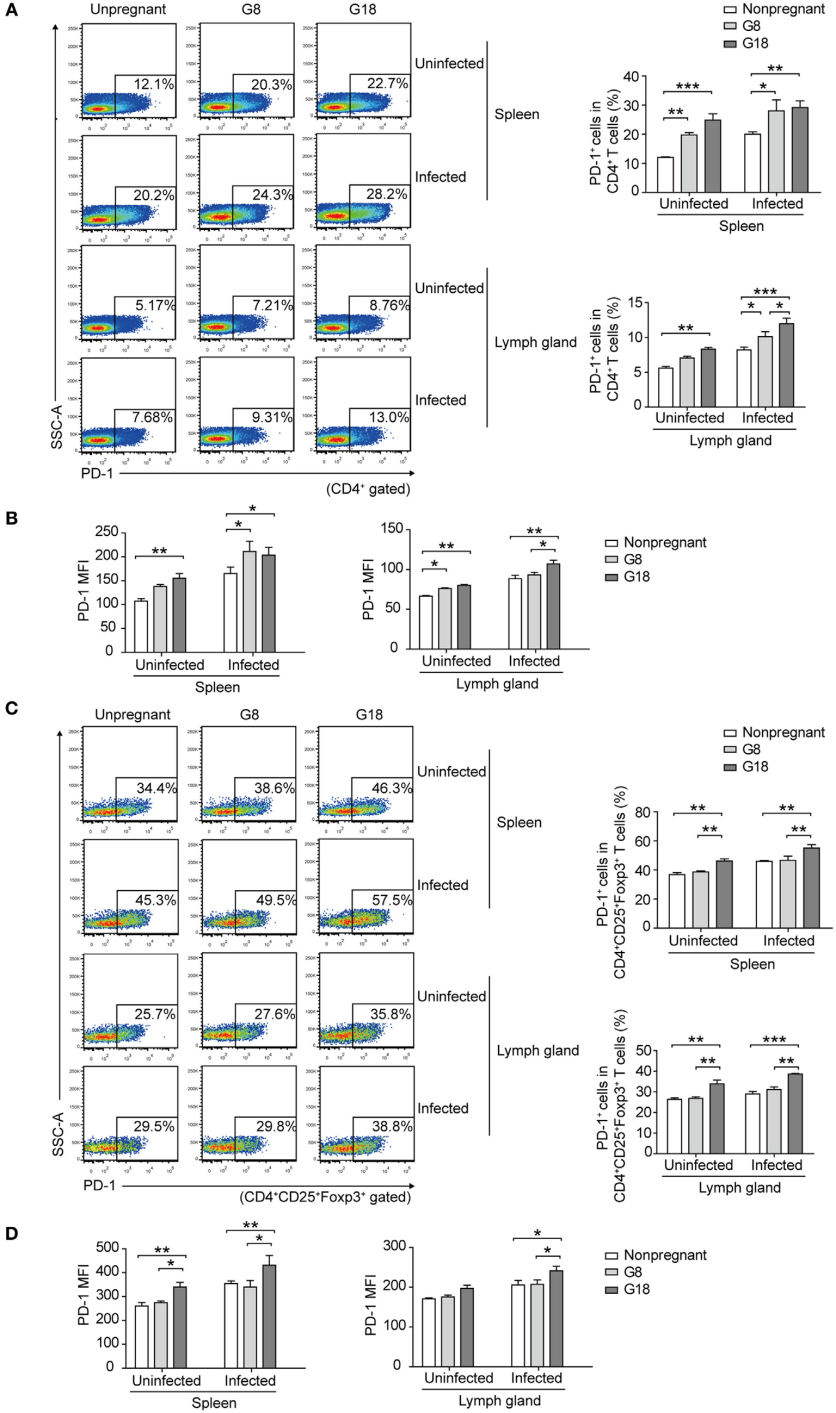
*Toxoplasma gondii* infection in early pregnancy induced lower levels of programmed death-1 (PD-1) expression on Tregs than that in late pregnancy. The percentage of PD-1^+^ cells in CD4^+^ T cells **(A)** and the mean fluorescence intensity (MFI) of PD-1 expression on CD4^+^ T cells **(B)** in spleens and inguinal lymph glands of the nonpregnant and pregnant mice (G8, G18) with *T. gondii* tachyzoite or PBS injection, respectively. The gating strategy for the identification of the mouse PD-1^+^CD4^+^ T cell population and the isotype control are shown in Figure S1B in Supplementary Material. Data are expressed as the means ± SD of five mice for each group from one experiment representative of two independent experiments. Significance was analyzed by one-way ANOVA. ***P < 0.001, **P < 0.01, *P < 0.05. The percentage of PD-1^+^ cells in CD4^+^CD25^+^Foxp3^+^ T cells **(C)** and the MFI of PD-1 expression on CD4^+^CD25^+^Foxp3^+^ T cells **(D)** in spleens and inguinal lymph glands from the mice as mentioned above. The gating strategy for the identification of the mouse PD-1^+^CD4^+^CD25^+^Foxp3^+^ T cell population and the isotype control are shown in Figure S1B in Supplementary Material. Data are expressed as the means ± SD of five mice for each group from one experiment representative of two independent experiments. Significance was determined by one-way ANOVA. ***P < 0.001, **P < 0.01, *P < 0.05.

To observe the changes of systemic immune regulation in pregnant mice infected with *T. gondii* in different stages of gestation, we analyzed the apoptotic percentage and PD-1 expression of regulatory T cells in spleens and inguinal lymph glands of the sacrificed mice as mentioned above by flow cytometry. The similar results were observed in spleen and inguinal lymph glands. The percentage of apoptotic Tregs showed no significant difference between early and late pregnancy in uninfected mice (Figure 3). However, after infection of *T. gondii*, the apoptotic rate of Tregs in early pregnancy was much higher than that in late pregnancy. This result suggested that the decreased expression of Foxp3 in early pregnancy was due to the increased apoptotic rate of Tregs. Besides, the expression of PD-1 on CD4^+^ T cells and Tregs was evaluated (Figure 4). The frequency of PD-1^+^ cells in CD4^+^ T cells did not exhibit any significant difference between early pregnancy and late pregnancy group in spleens (*P* > 0.05) and only showed a statistical difference between early pregnancy and late pregnancy group in lymph glands of *T. gondii*-infected mice (*P* < 0.05). However, the frequency of PD-1^+^ cells in Tregs in early pregnancy was much lower than that in late pregnancy in both *T. gondii*-infected and -uninfected group (*P* < 0.01). Similar patterns were observed when PD-1 expression was analyzed by mean fluorescence intensity (MFI) in CD4^+^ T cells and Tregs. These results further confirmed that *T. gondii* infection in late pregnancy induced a generalized decreased apoptotic rate and an increased PD-1 expression of Tregs compared to that in early pregnancy.

### E2 Protects Tregs Against Apoptosis and Upregulates the PD-1 Expression on Tregs

It has been shown that E2 drives the expansion of the CD4^+^CD25^+^ regulatory T cell compartment (30). On the ground of the reported regulation action of E2 on Tregs, we detected the level of E2 in mouse serum at different time points of pregnancy. The serum from pregnant mice at different gestation days (G0, G5, G6, G7, G8, G9, G14, G15, G16, G17, and G18) was collected. E2 in the serum was measured by ELISA. The result showed that the level of E2 in mouse serum gradually increased with the development of pregnancy (Figure S2 in Supplementary Material). This is consistent with normal pregnant women whose E2 level peaks in ante partum. This result suggested that the downregulated apoptotic rate and the upregulated PD-1 expression of Tregs in late pregnant group might be related with the increased level of E2 in late pregnancy.

**Figure 5:**
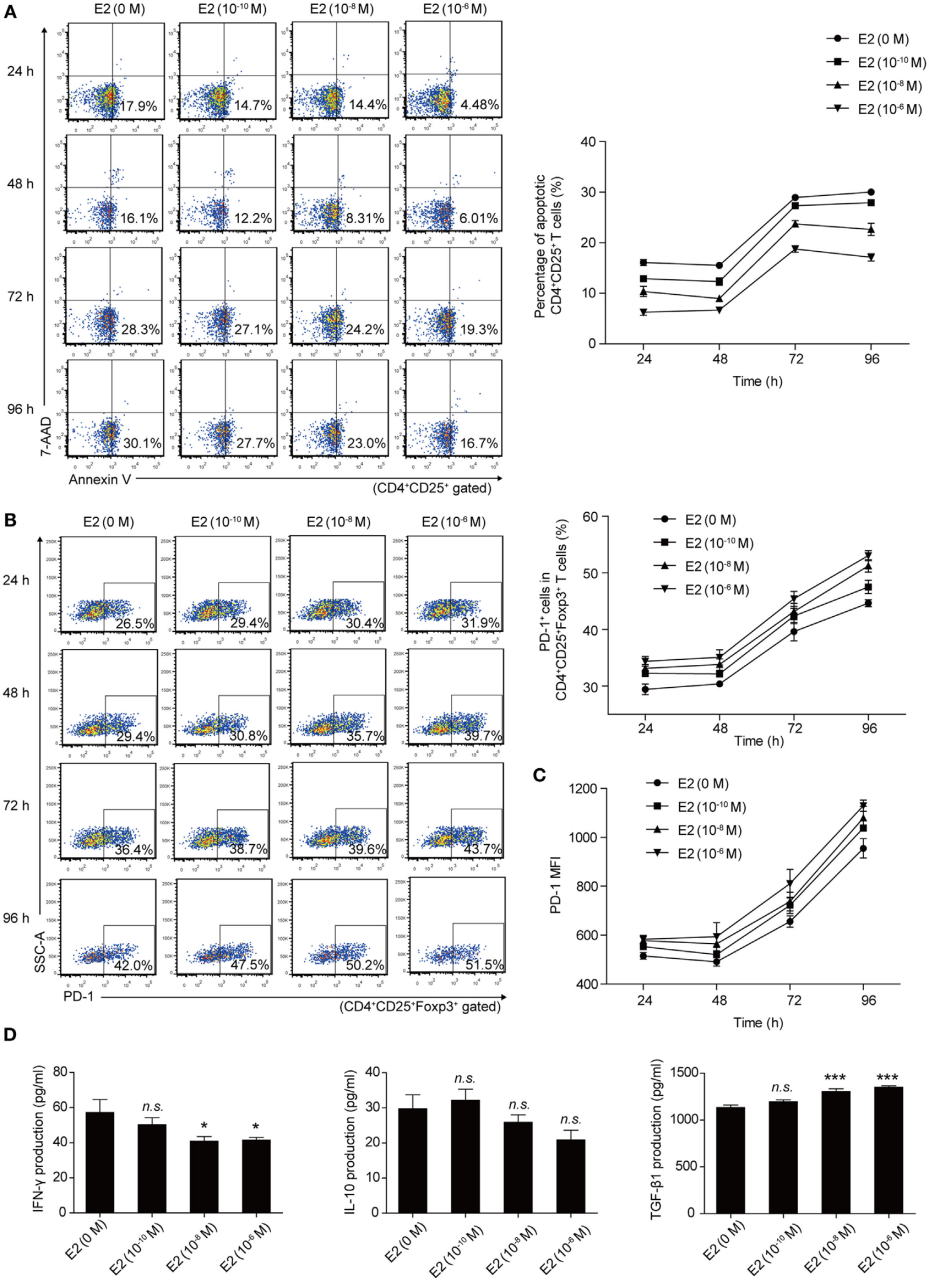
Estradiol (E2) inhibited the apoptosis of Tregs and enhanced the programmed death-1 (PD-1) expression on Tregs in a dose-dependent manner. **(A)** Splenocytes from C57BL/6 mice were treated with E2 ranging from 10^−10^ to 10^−6^ M for 24, 48, 72, and 96 h, respectively. The apoptotic rate of Tregs was estimated by Annexin V/7AAD analysis gated on CD4^+^CD25^+^ T cells. Data are presented as the means ± SD (*n* = 6). **(B)** Splenocytes were treated with E2 as mentioned above. The percentage of PD-1^+^ cells in CD4^+^CD25^+^Foxp3^+^ T cells was evaluated by flow cytometry. Data are expressed as the means ± SD (*n* = 6). **(C)** Mean fluorescence intensity (MFI) of PD-1 expression on CD4^+^CD25^+^Foxp3^+^ T cells. Data are presented as the means ± SD (*n* = 6). **(D)** The levels of IFN-γ, IL-10, and TGF-β1 secretion by mouse splenocytes stimulated with E2 ranging from 10^−10^ to 10^−6^ M for 96 h. Data are represented as the means ± SD (*n* = 6). Significance was analyzed using one-way ANOVA. ***P < 0.001, *P < 0.05, n.s. *P* > 0.05.

Then, we analyzed the apoptotic rate and PD-1 expression of Tregs with the *in vitro* treatment of E2. Mouse splenocytes were exposed to several doses of E2 (10^−10^, 10^−8^, and 10^−6^ M), and the apoptotic rate and the PD-1 expression of Tregs were detected at 24, 48, 72, and 96 h, respectively, by flow cytometry. The results showed that the *in vitro* administration of E2 decreased the apoptotic rate of Tregs and boosted not only the percentage of PD-1^+^ cells in Tregs but also the MFI of PD-1 expression on Tregs in a dose-dependent manner (Figures 5A–C). Such an effect is more obvious especially after 48 h. This result confirmed that E2 could enhance the expression of PD-1 on Tregs and further revealed that *in vitro* administration of E2 could inhibit the apoptosis of Tregs in a dose-dependent manner. In addition, to evaluate the activity of Tregs, inflammatory cytokine (IFN-γ) and anti-inflammatory cytokines (IL-10, TGF-β1) secreted by splenocytes were measured after 96-h *in vitro* administration of E2. E2 at relatively high levels (10^−8^ and 10^−6^ M), but not low concentration (10^−10^ M), decreased the production of IFN-γ and increased the production of TGF-β1 secreted by splenocytes. However, the levels of IL-10 did not change significantly (Figure 5D, *P* > 0.05). Since E2 did not decrease the percentage of CD4^+^ cells in lymphocytes (Figure S3 in Supplementary Material), the declined expression of IFN-γ was not correlated with the number of CD4^+^ T cells. The results indicated in part that E2 could enhance the suppressive role of regulatory cells.

### The Anti-Apoptotic Effect of E2 on Tregs and E2-Induced PD-1 Expression are Exclusively Dependent on ERα

**Figure 6:**
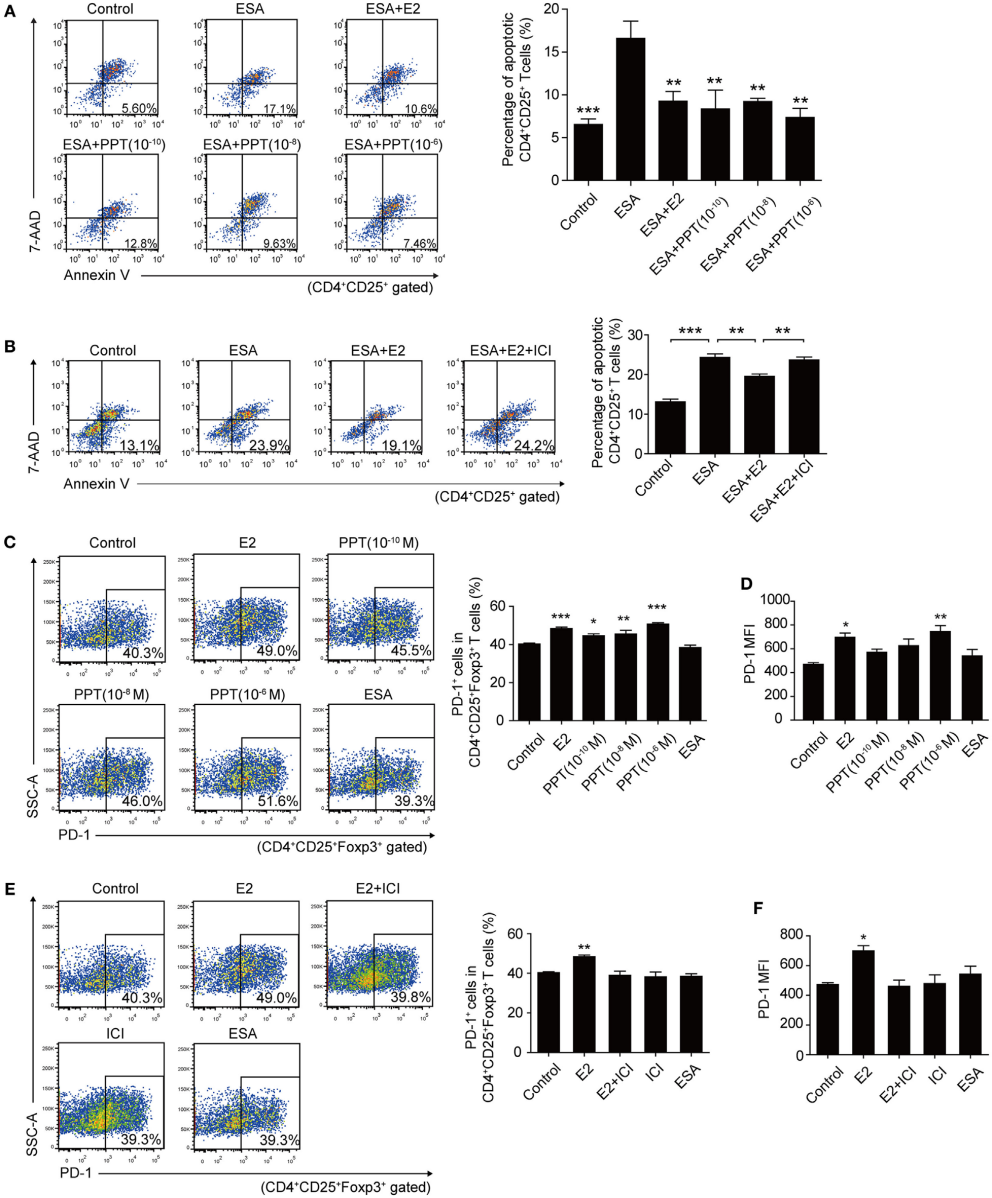
Estradiol (E2) inhibited the apoptosis of Tregs and enhanced the programmed death-1 (PD-1) expression on Tregs through ERα. **(A)** Splenocytes from C57BL/6 mice were treated with OVA (Control), excreted–secreted antigens (ESA), ESA and E2, ESA and ERα agonist (PPT) with different concentrations (10^−10^, 10^−8^, and 10^−6^ M), respectively. The apoptotic rate of CD4^+^CD25^+^ T cells was evaluated by flow cytometry. Data are expressed as the means ± SD (*n* = 4) and are representative of two independent experiments. Significance was determined by one-way ANOVA. ***P < 0.001, **P < 0.01. **(B)** Splenocytes from C57BL/6 mice were treated with OVA (control), ESA, ESA and E2, and ESA together with E2 and ER antagonist ICI 182,780 (ICI), respectively. The apoptotic rate of CD4^+^CD25^+^ T cells was evaluated by flow cytometry. Data are expressed as the means ± SD (*n* = 4) and are representative of two independent experiments. Significance was determined by one-way ANOVA. ***P < 0.001, **P < 0.01. **(C)** Representative dot plots and the bar graph show the frequency of PD-1^+^ cells in Tregs after treating with E2, PPT with different concentrations (10^−10^, 10^−8^, and 10^−6^ M) and ESA, respectively. Each bar indicates the mean value ± SD (*n* = 4) and is representative of two independent experiments. Significance was determined by one-way ANOVA. ***P < 0.001, **P < 0.01, *P < 0.05. **(D)** The mean fluorescence intensity (MFI) of PD-1 expression on CD4^+^CD25^+^Foxp3^+^ T cells. Data are expressed as the means ± SD (*n* = 4) and are representative of two independent experiments. Significance was determined by one-way ANOVA. **P < 0.01, *P < 0.05. **(E)** Representative dot plots and the bar graph show the frequency of PD-1^+^ cells in Tregs after treating with E2, ICI, E2 and ICI, and ESA, respectively. Data are expressed as the means ± SD (*n* = 4) and are representative of two independent experiments. Significance was determined by one-way ANOVA. **P < 0.01. **(F)** The MFI of PD-1 expression on CD4^+^CD25^+^Foxp3^+^ T cells. Data are expressed as the means ± SD (*n* = 4) and are representative of two independent experiments. Significance was determined by one-way ANOVA. *P < 0.05.

There are two known intracellular ERs, ERα and ERβ, which are expressed in a tissue-specific manner. However, CD4^+^ T cells express detectable ERα mRNA, but not ERβ mRNA (26). Thereby, we sought to detect the role of ERα in inhibiting the apoptosis of Tregs and enhancing the expression of PD-1 on Tregs. To assess whether the protection of E2 against apoptosis of Tregs is dependent upon ERα, 1,3,5-Tris(4-hydroxyphenyl)-4-propyl-1H-pyrazole (PPT), ERα agonist, and fulvestrant (ICI 182,780), ER antagonist, were, respectively, added to the cultured splenocytes. The treatment of PPT could significantly decrease the *T. gondii* ESA-induced-apoptotic rate of Tregs when the level of PPT reached 10^−10^ M (Figure 6A, *P* < 0.01). Simultaneously, the treatment of ICI 182,780 and E2 failed to decrease the apoptotic rate of Tregs. And the treatment only with E2 protected Tregs against *T. gondii* ESA-induced apoptosis (Figure 6B). The expression of PD-1 on Tregs was also analyzed after treating with PPT and ICI 182,780. When the level of ERα agonist PPT reached 10^−6^ M, the frequency of PD-1^+^ cells in Tregs, as well as the relative PD-1 MFI, markedly increased (Figures 6C,D). Moreover, ER antagonist ICI 182,780 could effectively inhibit the E2-mediated upregulation of PD-1 expression on Tregs (Figures 6E,F).

### The Simulated Mid-Pregnancy Level of E2 in Nonpregnant Mice Alleviated the *T. gondii* Infection-Induced Apoptosis of Tregs by Enhancing the Expression of Bcl-2 and Potentiated the PD-1 Expression on Tregs

**Figure 7:**
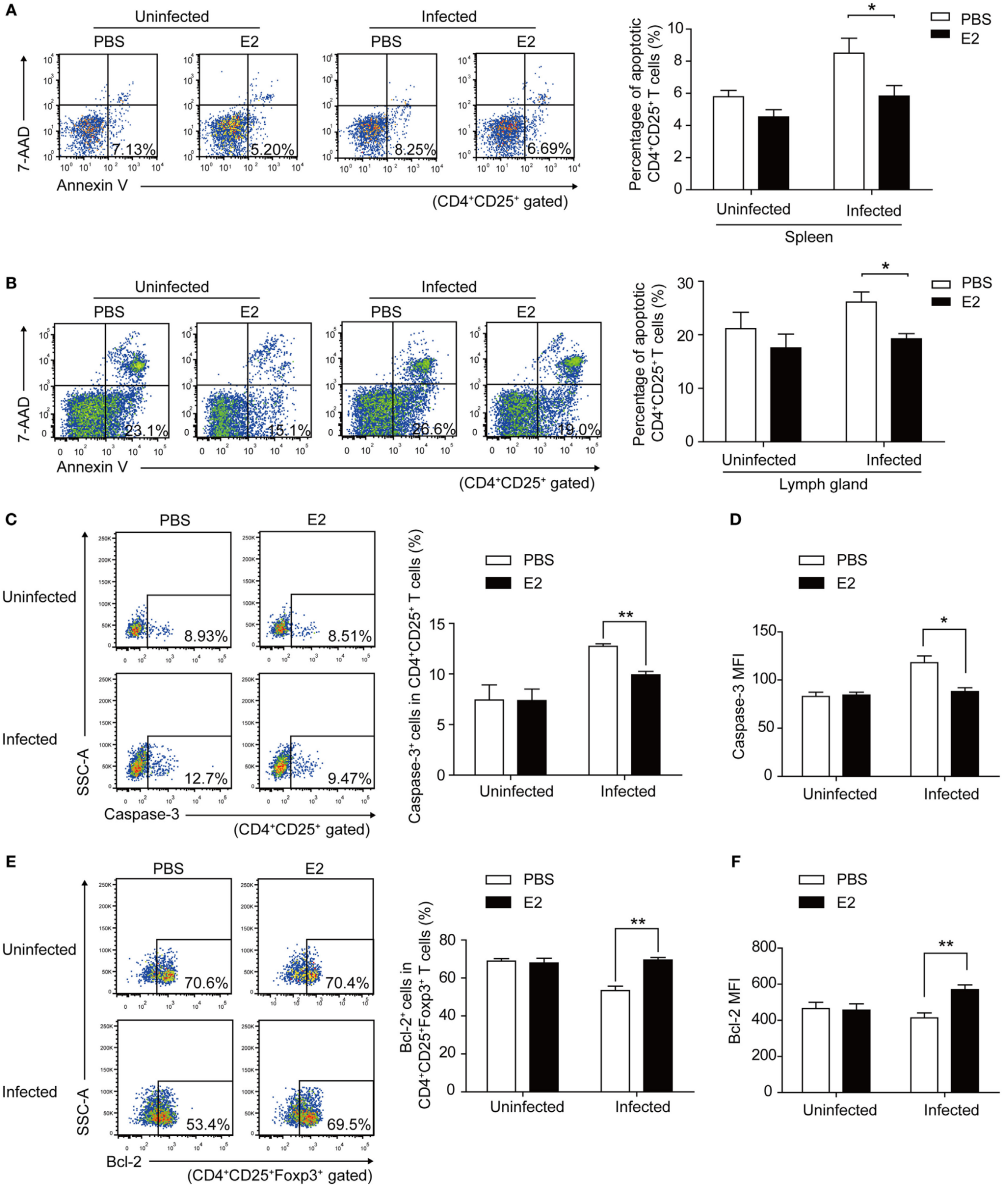
Estradiol (E2) *in vivo* administration in nonpregnant mice decreased the *Toxoplasma gondii* infection induced apoptosis rate of Tregs by enhancing the expression of Bcl-2. After a 2-week injection of E2 or PBS (control) into nonpregnant mice, the percentage of apoptotic Tregs in spleens **(A)** and inguinal lymph nodes **(B)** of mice was evaluated by flow cytometry. Error bars represent the means ± SD of six mice for each group from one experiment representative of two independent experiments. Significance was determined by the two-tailed Student’s *t*-test. *P < 0.05. **(C–F)** The splenocytes were isolated from nonpregnant mice with E2 *in vivo* administration. The frequency of caspase-3^+^ cells in CD4^+^CD25^+^ T cells **(C)** and the mean fluorescence intensity (MFI) of caspase-3 expression in CD4^+^CD25^+^ T cells **(D)** were measured by flow cytometric analysis. The gating strategy for the identification of the mouse caspase-3^+^CD4^+^CD25^+^ T cell population is shown in Figure S5A in Supplementary Material. Data are presented as the means ± SD of six mice for each group from one experiment representative of two independent experiments. Significance was determined by the two-tailed Student’s *t*-test. **P < 0.01, *P < 0.05. The percentage of Bcl-2^+^ cells in CD4^+^CD25^+^Foxp3^+^ T cells **(E)** and the MFI of Bcl-2 expression in CD4^+^CD25^+^Foxp3^+^ T cells **(F)** were measured by flow cytometric analysis. The gating strategy for the identification of the mouse Bcl-2^+^CD4^+^CD25^+^Foxp3^+^ T cell population is shown in Figure S5B in Supplementary Material. Each bar indicates the mean value ± SD of six mice for each group from one experiment representative of two independent experiments. Significance was determined by the two-tailed Student’s *t*-test. **P < 0.01.

To investigate whether E2 *in vivo* could protect Tregs against *T. gondii*-induced apoptosis and upregulate the expression of PD-1, we subcutaneously injected E2 into nonpregnant mice to simulate a high serum level of E2 in nonpregnant mice. After 2-week injection of E2, the level of E2 in the serum of nonpregnant mice was up to the level of E2 in mid-pregnancy (Figure S4 in Supplementary Material). The percentage of apoptotic Tregs in spleens and inguinal lymph nodes significantly decreased in E2 injection groups than that in PBS injection groups after *T. gondii* infection (*P* < 0.05, Figures 7A,B). Then, we further explored the possible mechanism of this anti-apoptotic effect. Caspase-3 plays an irreplaceable role in apoptosis. Therefore, we firstly detected the expression of caspase-3 on Tregs in the splenocytes isolated from nonpregnant mice with a 2-week E2 *in vivo* administration. We found that *T. gondii* infection increased the frequency of caspase-3^+^ cells in Tregs and the MFI of caspase-3 expression in Tregs, while the treatment of E2 alleviated the expression of caspase-3 (Figures 7C,D). Besides, we measured the expression of Bcl-2 on Tregs, which served as a distinct negative regulator of apoptosis (31). The result showed that E2 could upregulate the frequency of Bcl-2^+^ cells in Tregs as well as Bcl-2 MFI in *T. gondii* infection group (Figures 7E,F, *P* < 0.01). These results indicated that E2 had the capacity to inhibit the apoptosis of Tregs by increasing the expression of Bcl-2.

**Figure 8:**
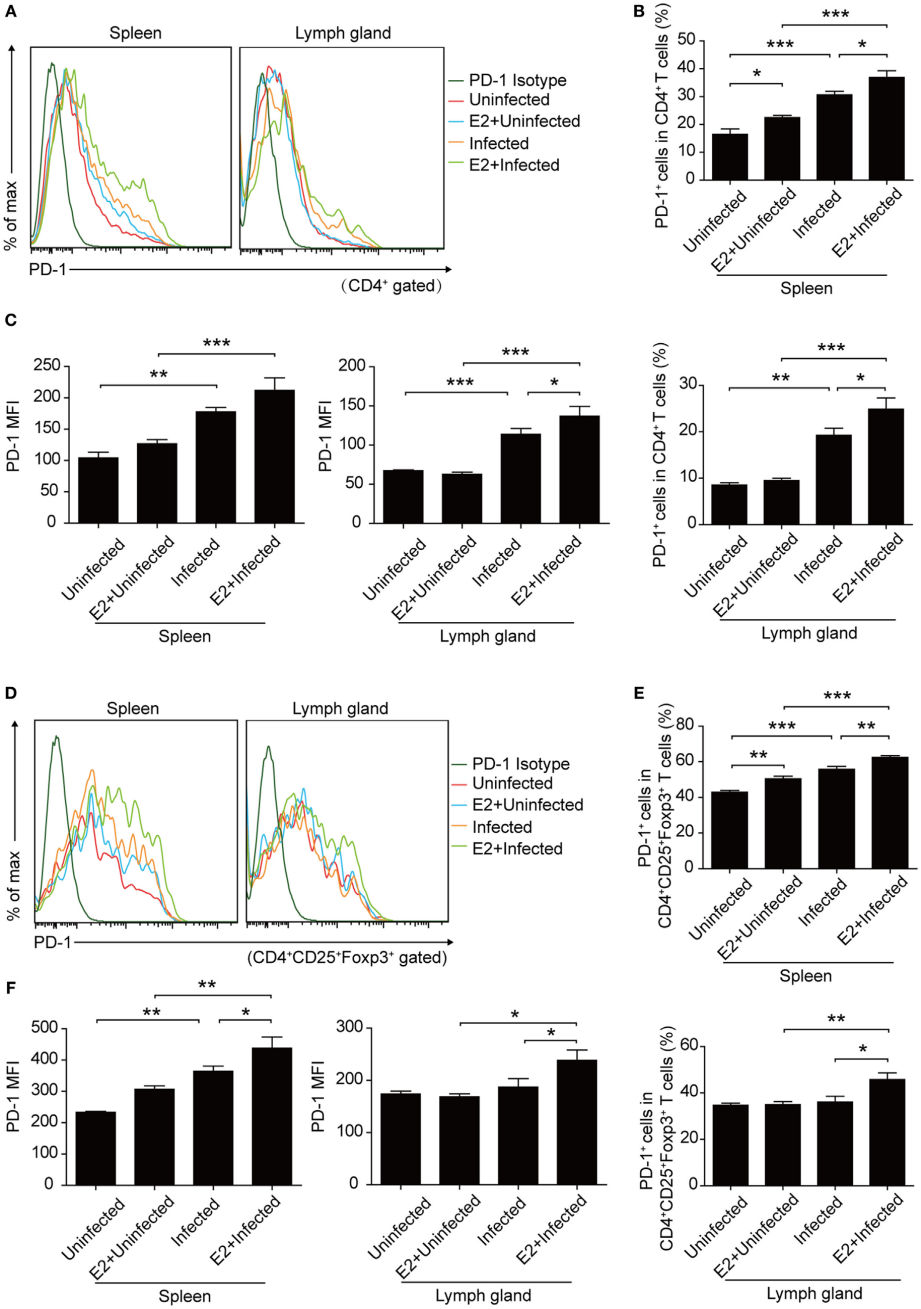
Estradiol (E2) *in vivo* administration in nonpregnant mice increased the expression of programmed death-1 (PD-1). **(A)** Overlay of representative histograms showing PD-1 expression on CD4^+^ T cells from spleens and inguinal lymph nodes of mice with E2 *in vivo* administration. The related dot plots were shown in Figure S6A in Supplementary Material. The percentage of PD-1^+^ cells in CD4^+^ T cells **(B)** and the mean fluorescence intensity (MFI) of PD-1 expression on CD4^+^ T cells **(C)** from spleens and inguinal lymph nodes of mice with E2 *in vivo* administration. Data are represented as the means ± SD of six mice for each group from one experiment representative of two independent experiments. Significance was determined by one-way ANOVA. ***P < 0.001, **P < 0.01, *P < 0.05. **(D)** Overlay of representative histograms showing PD-1 expression on CD4^+^CD25^+^Foxp3^+^ T cells from spleens and inguinal lymph nodes of mice with E2 *in vivo* administration. The related dot plots were shown in Figure S6B in Supplementary Material. The percentage of PD-1^+^ cells in CD4^+^CD25^+^Foxp3^+^ T cells **(E)** and the MFI of PD-1 expression on CD4^+^CD25^+^Foxp3^+^ T cells **(F)** from spleens and inguinal lymph nodes of mice as mentioned above. Data are represented as the means ± SD of six mice for each group from one experiment representative of two independent experiments. Significance was determined by one-way ANOVA. ***P < 0.001, **P < 0.01, *P < 0.05.

Moreover, after E2 *in vivo* administration, both the frequency and the MFI of PD-1 expression on CD4^+^ T cells and Tregs increased to a similar level as that in pregnancy, compared with PBS injection controls (Figure 8). This result further confirmed that the level of E2 in mid-pregnancy could enhance the PD-1 expression.

## Discussion

A perplexing phenomenon in congenital toxoplasmosis is that the severity of *T. gondii*-induced adverse pregnancy outcome is closely related with the timing of primary infection during pregnancy. *T. gondii* can cause pregnant women to undergo miscarriage and apoptosis of placental cells in early pregnancy. However, infections during late gestation generate extremely low rate of ill newborns (4). In this study, we conducted *in vitro* and *in vivo* experiments to reveal the underlying mechanism of this phenomenon. Our results showed that a high level of E2 in late pregnancy alleviated the *T. gondii* infection-induced apoptosis of Tregs and potentiated the PD-1 expression on Tregs, thus helping maintain the immune balance and improve the pregnancy outcome. These results further shed light on the mechanism of maternal resistance to infection-induced miscarriage and provide new insights into the clinical prevention and treatment of adverse pregnancy outcomes caused by *T. gondii*.

Tregs are one subpopulation of CD4^+^ T cells which play a vital role in normal pregnancy. In humans, low levels of circulating Tregs have been served as a predictive signal of a risk of miscarriage in pregnant woman with a history of abortion (32, 33). Among several subsets of Tregs, PD-1^+^ Tregs mainly exerted the immunosuppressive function (34). It is suggested that Tregs might play a role of immunosuppression through the PD-1/PD-L1 pathway. Our results demonstrated that in early pregnancy, the infection of *T. gondii* induced apoptosis of Tregs in pregnant mice, which led to abortion. However, infection with *T. gondii* tachyzoites in late pregnancy induced a lower apoptotic rate of Tregs and a higher percentage of PD-1^+^ Tregs than that in early pregnancy, which corresponded to invisible necrosis and hemorrhage in fetuses and placentas and lower abortion rates (Figure 1). This finding revealed that Tregs involved in the *T. gondii* infection induced abortion.

Estradiol exerts great influence on the maintenance of immune balance and normal pregnancy (35). In this study, we reported that the level of E2 in mice gradually increased during pregnancy. The level of E2 in late pregnancy is much higher than that in early pregnancy. Thus, we speculated that E2 might participate in the mechanism by which *T. gondii* tachyzoites failed to induce the abortion in late pregnancy. We tested the capacity of E2 in inhibiting the apoptosis of Tregs and potentiating the expression of PD-1. The treatment with E2 or ERα agonist could notably lessen the *T. gondii* ESA-induced apoptosis of Tregs and reinforce PD-1 expression on Tregs in a dose-dependent manner, while treatment with E2 and ER antagonist together failed to induce such a phenomenon of those cells (Figures 5 and 6). E2 *in vivo* administration in nonpregnant mice also decreased the *T. gondii* infection-induced apoptosis of Tregs, and the moderated apoptosis of Tregs was attributed to the upregulated expression of Bcl-2 (Figure 7). Importantly, this result further confirmed that the level of E2 in middle pregnancy could attenuate the *T. gondii*-induced apoptosis of regulatory T cells. Moreover, *in vivo* E2 administration in nonpregnant mice also upregulates the frequency of PD-1^+^ cells in total Tregs to a similar level as that in pregnancy, suggesting that high levels of E2 during pregnancy help maintain fetal tolerance. Together, E2 could modulate the apoptosis and PD-1 expression of Tregs *via* ERα. These results highlight a potential use of E2, or its analogs, to manipulate Treg function.

Although PD-1 is known to trigger T cell exhaustion, the relationship between PD-1 expression and cell apoptosis is still ill-defined. PD-1/PD-L1 signaling pathway has different effects on T cells, such as downregulation of effector T cell levels, induction of apoptosis of tumor-infiltrating T cells, and promoting CD4^+^ T cell differentiation into suppressive Foxp3^+^ Tregs (36). In a mouse model of T cell lymphoma, a homo- or heterozygous deletion of PD-1 allows unrestricted T cell growth and leads to the rapid development of highly aggressive lymphomas *in vivo* (37). PD-1 can induce the apoptosis of CD8^+^ T cells. Horton et al. found that tumor antigen-specific CD8^+^ T cells (TILs) apoptosis was only seen in activated TILs expressing PD-1, while apoptosis was not observed in the PD-1^−^ TIL population (38). Guida et al. found that rats exposed to gamma radiation exhibited an increased exhaustion of T lymphocytes *via* downregulation of Bcl-2 expression and upregulation of PD-1, Bax, and caspase-3 expression, which sensitized these cells to apoptosis (39). However, in our study, we found that the expression of PD-1 simultaneously upregulated with a decreased apoptotic rate of Tregs after E2 treatment in *T. gondii* infection model. Similarly, in chronic graft-versus-host disease model, PD-1-deficient Tregs showed proapoptotic with a higher Fas and a lower Bcl-2 expression (40). Toor et al. found that PD-1 was expressed mainly on CD4^+^CD25^+^ T cells, and anti-PD-1 antibody, pembrolizumab, had a greater effect on PD-1 expression on CD4^+^CD25^−^ T cells, compared to CD4^+^CD25^+^ T cells (36). Therefore, given the fact that different subpopulations of T cells exhibit diverse functions and characteristics, we can draw the conclusion that the role of PD-1 and its relationship with cell apoptosis are not immutable in different disease models.

Sex hormones, such as E2, play a crucial and intricate role during pregnancy to mediate several aspects of the pregnancy process (41, 42). In the present study, the serum levels of E2 from pregnant mice were measured and they increased gradually over the entire course of pregnancy. This is consistent with the change curve of E2 during human pregnancy. However, to date, the research on the correlation between E2 and Tregs during pregnancy is quite limited. Only a few papers on the Foxp3 and E2 have been reported, and the correlation study on E2 and the apoptosis of Tregs is lacking. It was found that the percentage of Foxp3^+^ T cells in the peripheral blood of pregnancy woman with missed abortion was lower than that in normal pregnant and healthy nonpregnant controls, and the low levels of Tregs positively correlated with the serum concentrations of E2 (43). In our *T. gondii* infection model, E2 levels are also correlated with changes of Tregs, in both their apoptotic rate and PD-1 expression. This result suggested that E2 was a regulator of Tregs. It may control maternal tolerance through regulating Tregs. This phenomenon sheds light on the intricate working of hormone–immune system interaction in *T. gondii*-induced abnormal pregnancy. However, the mechanism of action for E2 in other cell types of the immune system needs further investigation.

In addition to *T. gondii*, other pathogens like cytomegalovirus (CMV), their intrauterine transmission, and clinical outcome are also largely depending on the timing of primary maternal infection during pregnancy (44–46). As for CMV infection in pregnant woman, the highest risk of severe symptoms in the fetus and newborn exists in the first trimester of pregnancy (44). To protect the mother and the fetus, the immune system has diverse coping strategies to deal with infection in different stages of gestation (29, 47). It is during early pregnancy when the maternal immune system is characterized by a reinforced network of recognition, repair, raising the alarm of invading pathogens: if necessary, to induce intense inflammatory response and protect the mother and the fetus from pathogens. During late pregnancy, without any doubt, the maternal immune system provides a unique suppressive immune condition that will modify the way the mother responds to the environment and the “foreign” fetus, facilitating and protecting the pregnancy (29). Therefore, this unique behavior during pregnancy explains why the immune system, especially Tregs, of pregnant women responds differently to the pathogen depending on the timing of primary infection during gestation.

In conclusion, this study provides evidence for the previously unclarified mechanism that the severity of *T. gondii*-induced adverse pregnancy outcomes depends on the timing of infection. The relationship between E2 and Tregs in the pathogenesis of *T. gondii*-induced abnormal pregnancy is revealed, which may facilitate the potential development of novel therapies for the treatment of infection factor-mediated abortion targeting the hormone–immune system pathway.

## Ethics Statement

This study received approval from the Animal Ethics Committee of Nanjing Medical University, and all animal experiments were conducted in accordance with the approved guidelines for the care and use of animals (Permit Number: NJMU/IACUC 1403005).

## Author Contributions

JQ, JW, and YW conceived the project. Experiments were designed by JQ and YW. JQ, RZ, YX, LW, KG, and HC performed cell and animal experiments. Statistical analyses were performed by LW and XL. All authors were involved in writing and revising the manuscript and approved the final version.

## Conflict of Interest Statement

The authors declare that the research was conducted in the absence of any commercial or financial relationships that could be construed as a potential conflict of interest.
