# Supplementary material for: Estradiol Attenuates the Severity of Primary Toxoplasma gondii Infection-Induced Adverse Pregnancy Outcomes Through the Regulation of Tregs in a Dose-Dependent Manner
Source: Front Immunol. 2018 May 18;9:1102. doi: 10.3389/fimmu.2018.01102 (PMC5968100; doi:10.3389/fimmu.2018.01102)
Supplement: Supplementary file 1 [file Data_Sheet_1.PDF]

Supplementary Material

Estradiol attenuates the severity of primary *Toxoplasma gondii* infection-induced adverse pregnancy outcomes through the regulation of Tregs in a dose-dependent manner

Jingfan Qiu, Rong Zhang, Yanci Xie, Lijuan Wang, Ke Ge, Hao Chen, Xinjian Liu, Jiangping Wu and Yong Wang*

*** Correspondence:** Yong Wang, email: yongwsh@njmu.edu.cn

1 Supplementary Figures

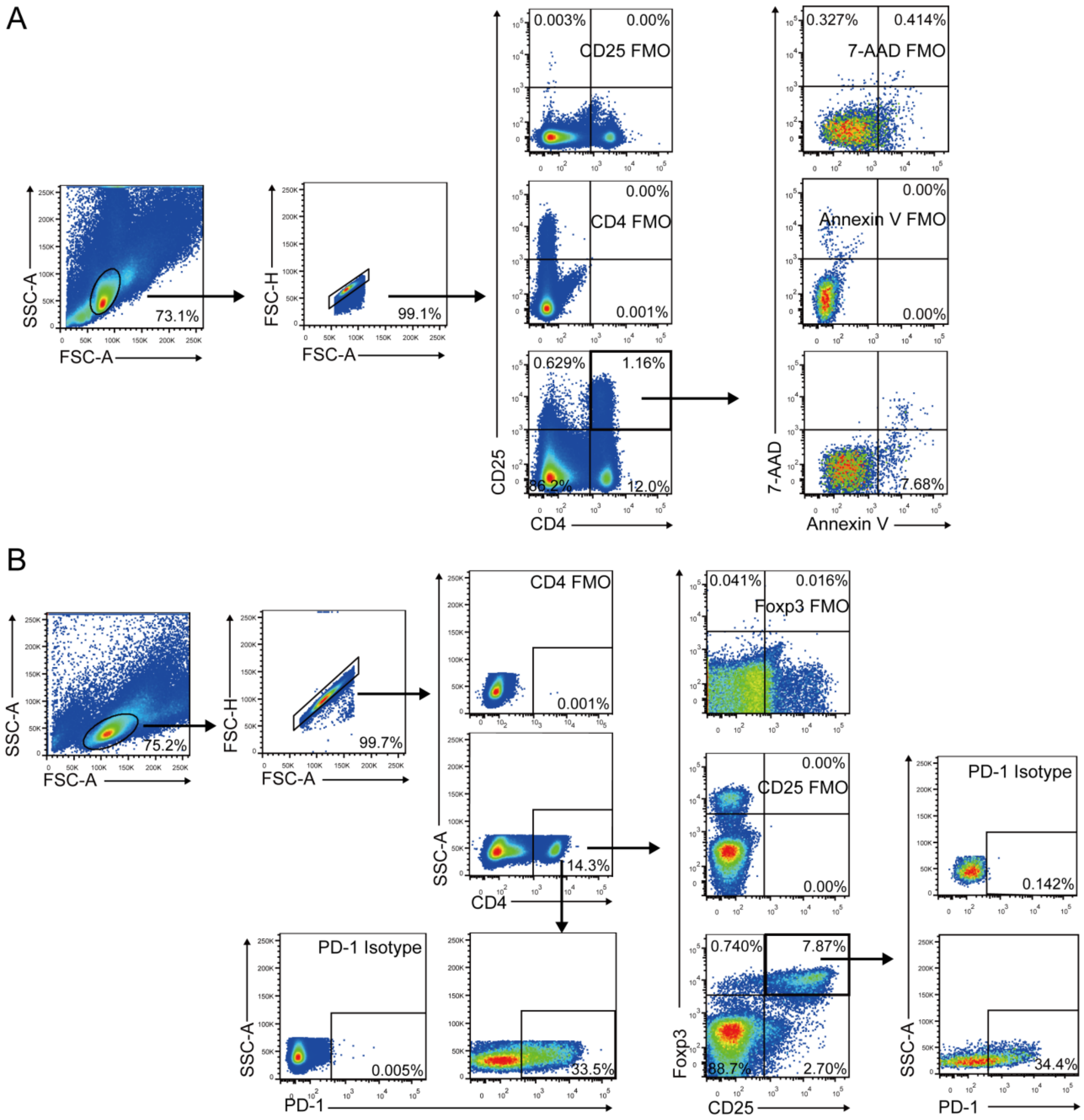

Supplementary Figure 1. FACS gating strategies. **(A)** Gating strategy for analysis of the apoptotic rate of $CD4^+CD25^+$ T cells (related to Figure 3A, 5A, 6A, 6B, 7A and 7B). **(B)** Gating strategy for analysis of the percentages of $PD\text{-}1^+$ cells in $CD4^+$ T cells or $CD4^+CD25^+Foxp3^+$ T cells (related to Figure 4A, 4C, 5B, 6C, 6E, 8A, 8D).

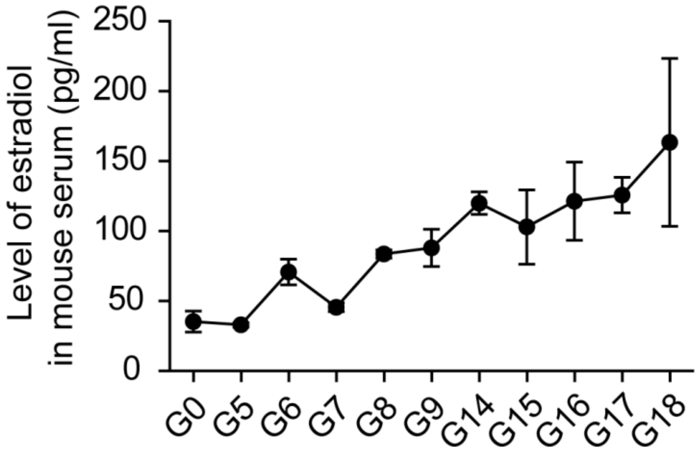

Supplementary Figure 2. The level of estradiol (E2) in mouse serum during pregnancy. The serum from pregnant mice during the entire course of pregnancy at gestation day 0, 5, 6, 7, 8, 9, 14, 15, 16, 17, 18 (G0, G5, G6, G7, G8, G9, G14, G15, G16, G17, G18) were collected. E2 in the serum was measured by ELISA. Data are represented as the means ± S.D. of 5 mice for each group from one experiment representative of two independent experiments.

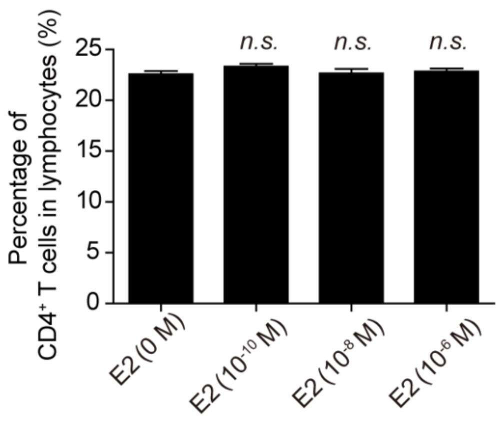

Supplementary Figure 3. The percentage of $CD4^+$ T cells in lymphocytes after 96 h-*in vitro* administration of E2 (10^{-10}, 10^{-8} and 10^{-6} M). Data are expressed as the means ± S.D. (n = 6). Significance was analysed using one-way ANOVA. *n.s.* $P > 0.05$ (compared with E2 0 M-group).

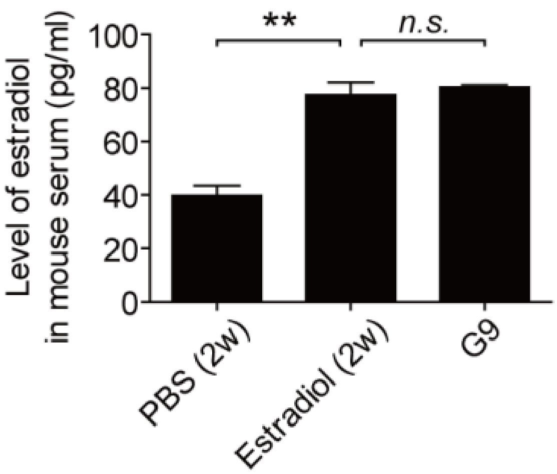

Supplementary Figure 4. The level of estradiol (E2) in mouse serum after E2 *in vivo* administration. After two-week injection of E2, the level of E2 in mouse serum was detected by ELISA. Data are represented as the means ± S.D. of 6 mice for each group from one experiment representative of two independent experiments. Significance was determined by one-way ANOVA. ** $P < 0.01$, *n.s.* $P > 0.05$.

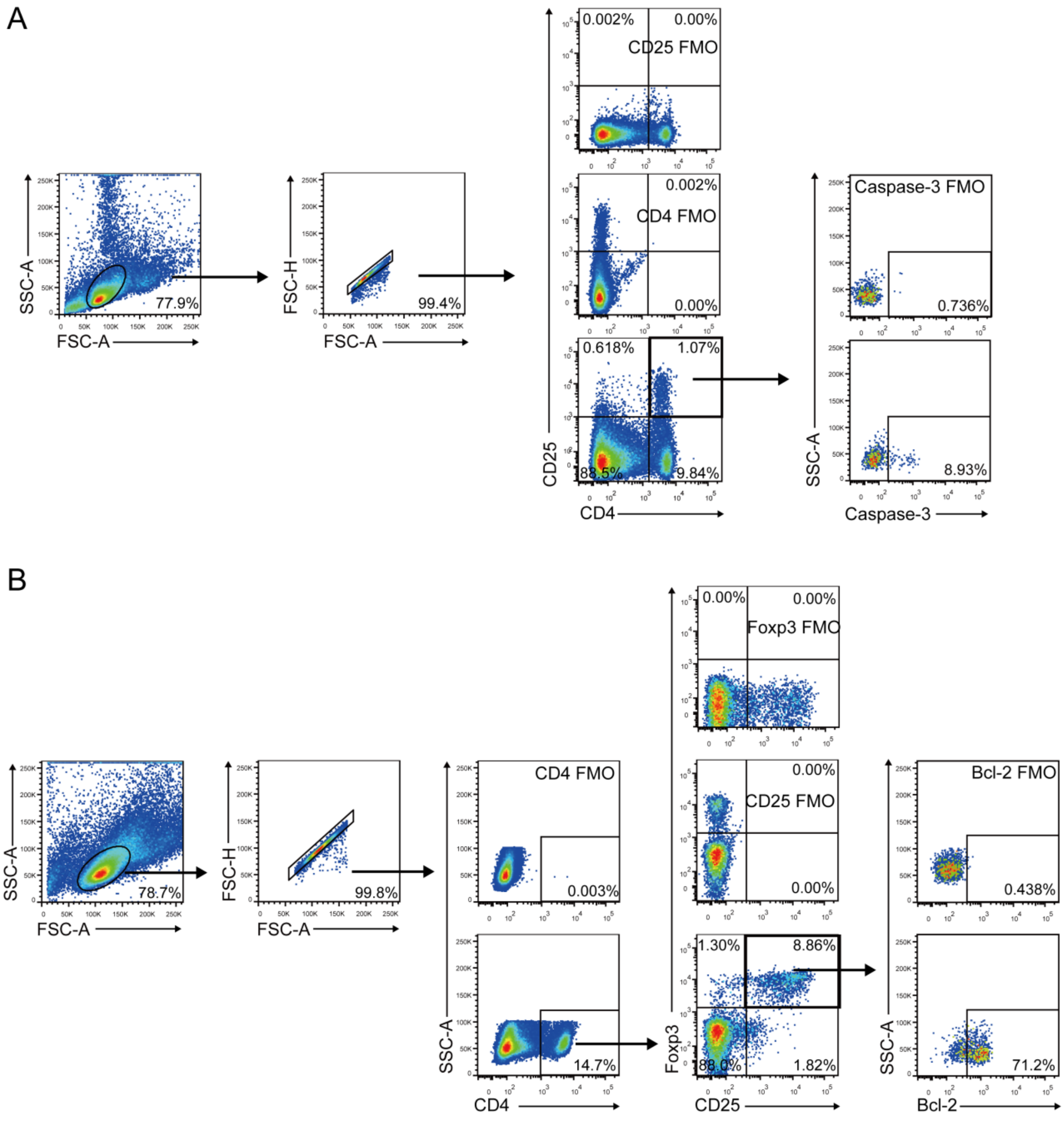

Supplementary Figure 5. FACS gating strategies. **(A)** Gating strategy for analysis of the percentages of Caspase-3$^+$ cells in CD4$^+$CD25$^+$ T cells (related to Figure 7C). **(B)** Gating strategy for analysis of the percentages of Bcl-2$^+$ cells in CD4$^+$CD25$^+$Foxp3$^+$ T cells (related to Figure 7E).

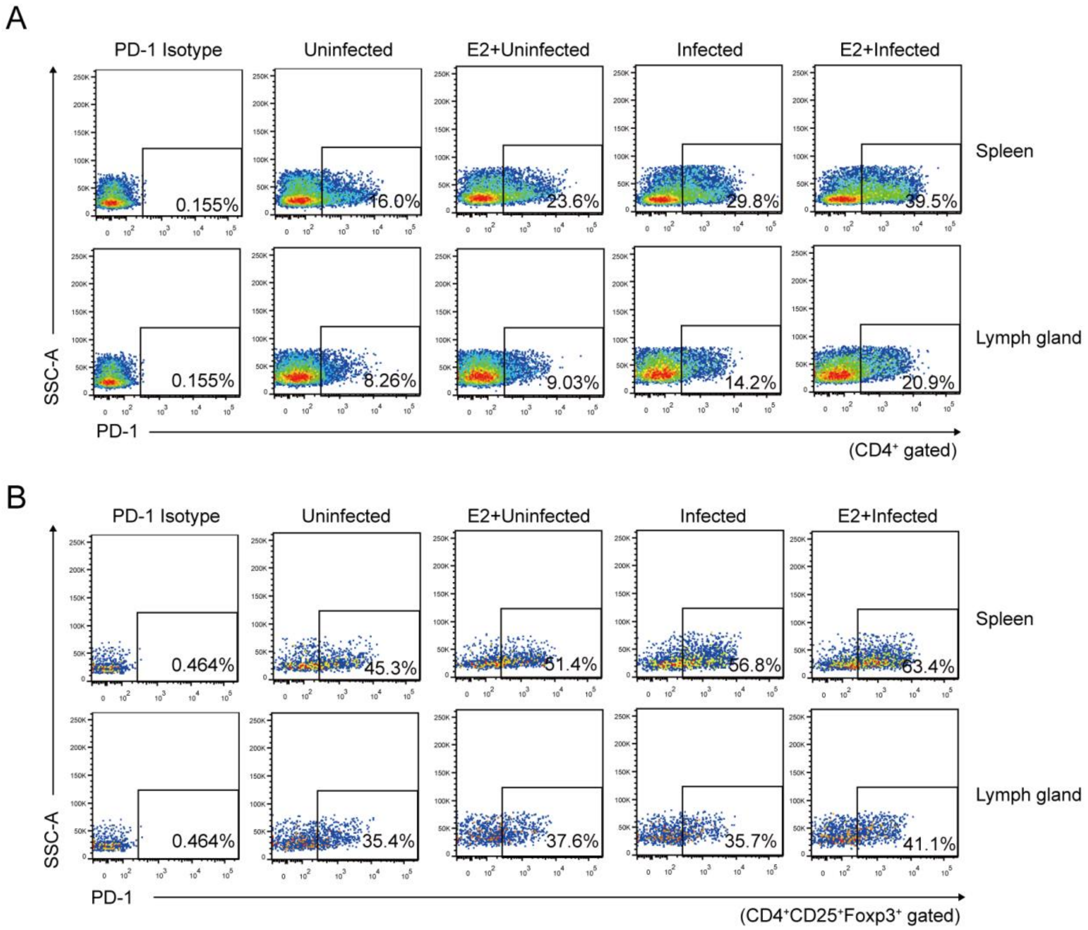

Supplementary Figure 6. Representative dot plots show the frequency of PD-1$^+$ cells in CD4$^+$ T cells **(A)** and CD4$^+$CD25$^+$Foxp3$^+$ T cells **(B)** from spleens and inguinal lymph nodes of mice with E2 *in vivo* administration (related to Figure 8A and 8D).
